# Comparison of Cutting Efficiency Between Natural and Synthetic Diamond-Coated Burs on Zirconia and Natural Teeth

**DOI:** 10.3390/ma18245623

**Published:** 2025-12-15

**Authors:** Da-Sol Kim, Sung-Bin An, Da-Hae Kim, Jung-Bo Huh, You-Jin Lee

**Affiliations:** 1 Department of Prosthodontics, School of Dentistry, Pusan National University, Yangsan 50612, Republic of Korea; rlaekthfsla@naver.com (D.-S.K.);; 2 Department of Prosthodontics, Dental Research Institute, Dental and Life Sciences Institute, School of Dentistry, Pusan National University, Yangsan 50612, Republic of Korea; 3 Department of Prosthodontics, Dental Clinic Center, Pusan National University Hospital, Busan 49241, Republic of Korea

**Keywords:** diamond burs, natural diamond, synthetic diamond, zirconia, cutting efficiency, dental materials, in vitro study

## Abstract

This in vitro study compared the cutting efficiency of natural diamond burs (NDB) and synthetic diamond burs (SDB) on zirconia and natural teeth. Two types of diamond rotary instruments with identical specifications (TR-13, Tapered Round End) were evaluated using 100 zirconia specimens and 100 extracted molars. Each bur was tested through 10 consecutive cutting cycles under standardized conditions (200,000 rpm, 2 N force, water coolant). Cutting efficiency was evaluated based on weight loss per unit time (mg/min). Surface characterization was performed using field-emission scanning electron microscopy (FE-SEM) and energy-dispersive X-ray spectroscopy (EDS). For zirconia cutting, NDB demonstrated significantly higher cutting efficiency than SDB at all cycles (*p* < 0.05), with 38.9% greater total material removal (115.97 ± 2.22 mg versus 83.46 ± 2.08 mg). NDB also exhibited lower wear rates (40.00% versus 49.68% reduction). For natural tooth cutting, no significant differences were observed between NDB and SDB (*p* > 0.05). EDS analysis showed that SDB exhibited greater carbon loss (13.46% versus 2.49%) and increased surface heterogeneity after repeated use. These findings indicate that natural diamond burs are superior for cutting high-hardness materials such as zirconia, while both bur types perform equivalently on natural teeth.

## 1. Introduction

Zirconia ceramics have become essential materials for fixed and implant-supported dental prostheses due to their superior mechanical properties and esthetic appearance [1,2]. The remarkable strength of zirconia originates from a transformation toughening mechanism involving a phase transition from the tetragonal to the monoclinic form [3,4]. Nevertheless, clinical complications such as secondary caries, pulpal pathology, or prosthetic failure often necessitate the removal or modification of existing zirconia restorations [5,6]. Because zirconia is considerably harder and has higher fracture toughness than feldspathic porcelain, removing it with conventional diamond rotary instruments often leads to reduced cutting efficiency and longer clinical procedures [7,8]. In contrast, the cutting of natural teeth requires a different balance between efficiency and control, as excessive heat or vibration can damage pulp tissue or surrounding structures. Therefore, understanding the cutting behavior of both zirconia and natural teeth is clinically important for optimizing rotary instrument performance [7,8].

To overcome these limitations, diamond rotary instruments specifically engineered for zirconia removal have been developed. Although some studies have shown that zirconia-specific diamond burs do not necessarily enhance cutting efficiency [9], they tend to produce smoother surface morphology with fewer micro-defects. Other research has reported that fine-grit rotary instruments (40–50 μm) provide superior cutting efficiency while minimizing visible damage and instrument wear [10,11]. Furthermore, repeated cutting has been observed to decrease efficiency regardless of bur design or diamond particle size [8,12]. Adequate load control and water cooling are therefore essential to maintain consistent performance and prevent thermal damage [11].

The diamonds used in rotary instruments can be categorized as natural or synthetic. Natural diamonds are typically Type Ia, containing nitrogen as an impurity, whereas synthetic diamonds are produced by either the High-Pressure High-Temperature (HPHT) method or the Chemical Vapor Deposition (CVD) method [13]. HPHT diamonds are synthesized from graphite using metallic catalysts such as iron, nickel, or cobalt under extreme conditions (5–6 GPa, 1300–1600 °C), generally yielding Type Ib diamonds with nitrogen and metal impurities [13]. In contrast, CVD diamonds are formed through vapor deposition using methane and hydrogen radicals at relatively low pressure and moderate temperature (700–1000 °C), producing Type IIa diamonds that may include silicon as a trace impurity.

Although synthetic diamonds often demonstrate superior hardness and thermal conductivity compared with natural diamonds, their quality can vary significantly depending on manufacturing conditions. Low-cost commercial synthetic diamonds may show degraded mechanical properties due to incomplete sintering, graphitization, or high impurity content [14].

Another crucial factor affecting cutting performance is the interface between the diamond particles and the instrument’s metal matrix. Interfacial reactions between diamond particles and nickel-based brazing materials can influence retention strength and durability [15]. Electroplating methods vary in their effectiveness, with conventional techniques showing greater diamond particle loss during repeated use compared to advanced bonding approaches [16]. These material-dependent characteristics directly influence both the efficiency and longevity of diamond rotary instruments [7,8,11,12]; however, systematic comparative studies on this topic remain limited.

Previous investigations have mainly focused on diamond particle size, instrument geometry, or product-specific performance. Comparisons based on the type of diamond—natural versus synthetic—are rarely reported. Moreover, while most studies have focused on zirconia cutting, few have included natural tooth structures as a comparative substrate.

Therefore, the purpose of this study is to compare the cutting efficiency of diamond burs fabricated with natural and synthetic diamonds and to evaluate their cutting characteristics and surface morphology on both zirconia and natural teeth.

## 2. Materials and Methods

### 2.1. Selection of Diamond Rotary Instruments

Two types of diamond rotary instruments with identical specifications (TR-13, Tapered Round End) produced by the same manufacturer were evaluated.

The experimental group consisted of natural diamond burs containing natural diamond particles bonded to the shank using a nickel sulfamate electroless-plating method, whereas the control group consisted of synthetic diamond burs containing synthetic diamond particles bonded using a nickel sulfate electroless-plating method.

All rotary instruments were unused prior to testing, and were stored under manufacturer-recommended conditions. The manufacturer’s name was anonymized to prevent potential conflicts of interest.

### 2.2. Specimen Preparation

#### 2.2.1. Zirconia Specimens

Pre-sintered zirconia discs (Zircos-E, shade A2, flexural strength 1100 MPa, translucency 20T; BIODEN, Hwain Sinso Material Co., Ltd., Osan, Republic of Korea) served as the substrate for zirconia cutting evaluation.

Rectangular specimens (32 × 16 × 6 mm) were designed and milled using the Trione-Z DIO CAD/CAM system (DIO Corporation, Busan, Republic of Korea).

A total of 100 zirconia specimens were fabricated, with 50 allocated to each bur group through random assignment to prevent allocation bias. Both sides of each specimen were utilized, yielding 100 cutting surfaces per group (*n* = 10 per cutting cycle). Sintering was performed following the manufacturer’s recommended protocol: heating to 1520 °C with intermediate holds at 900 °C (100 min) and 1250 °C (120 min), final hold at 1520 °C (120 min), and controlled cooling to room temperature.

#### 2.2.2. Natural Tooth Specimens

One hundred extracted maxillary and mandibular molars with intact enamel and no caries or restorations were collected from the Biobank of Pusan National University Dental Hospital (Korea Human Resource Bank Network, Project No. 2024ER050700).

All teeth were utilized within three months of extraction and stored in 0.1% thymol solution at 4 °C.

Prior to the experiment, teeth were cleaned with a hand scaler to remove debris and calculus. Each tooth was stabilized on baseplate wax during cutting. Fifty teeth were randomly assigned to each bur group to prevent allocation bias, and both buccal and lingual enamel surfaces were used to obtain 100 cutting surfaces per group (*n* = 10 per cutting cycle). Only sound enamel areas were selected as cutting targets. This study focused on enamel cutting for methodological reasons. When using extracted natural teeth, the outer enamel surface must be cut first before accessing underlying dentin. To minimize the influence of anatomical variation (enamel thickness, curvature, and prism orientation), teeth were selected based on similar size and the cutting was performed on the flat surface of the molars.

### 2.3. Cutting Efficiency Experiment

#### 2.3.1. Experimental Setup

**Figure 1:**
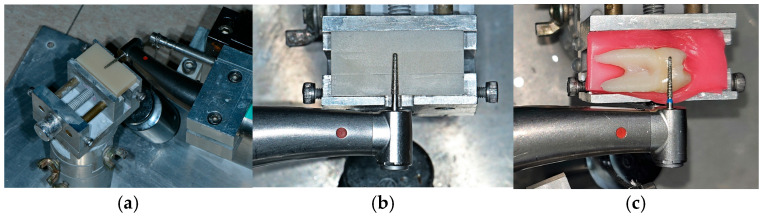
Custom-designed apparatus for standardized cutting [adapted from Kim et al., 2017 [7]. (**a**) Overview of the apparatus showing the fixed handpiece mounting system and specimen holder. (**b**) Full length of the cutting area of each diamond bur in contact with the zirconia block. (**c**) Full length of the cutting area of each diamond bur in contact with the tooth enamel surface.

A custom-designed apparatus, previously described and validated by Kim et al. [7], was used to maintain standardized cutting conditions throughout the experiment (Figure 1). The apparatus consisted of a fixed mounting system that secured the high-speed handpiece (T2 Line A 200L; Dentsply Sirona, York, PA, USA) at a consistent vertical orientation, ensuring perpendicular contact between the bur and specimen surface. The specimen holder was designed to maintain stable positioning during cutting and prevent lateral movement. This setup standardized the cutting angle, contact area, and vertical alignment across all 200 specimens, thereby minimizing operator-dependent variables.

Specimens were secured on a horizontal platform, with the diamond bur shank positioned perpendicularly. A constant force of 2 N was applied by attaching a 240 g weight at a distance of 5.5 cm from the rotational axis, following established protocols (Figure 1) [7].

#### 2.3.2. Cutting Protocol

1. **Pre-cutting measurement**: Each specimen was weighed using a precision electronic balance (PAG214C; Ohaus, Parsippany, NJ, USA; accuracy ± 0.001 g).
2. **Cutting conditions**: All burs were operated at 200,000 rpm under continuous water coolant (25 mL/min). The maximum speed was confirmed on the Elec LED display. The same high-speed turbine handpiece (T2 Line A 200L; Dentsply Sirona, York, PA, USA) was used throughout the experiment to ensure consistent conditions across all specimens.
  ∘ Zirconia specimens were cut for 60 s.
  ∘ Natural tooth enamel was cut for 30 s, corresponding to its lower hardness (≈3–4 GPa) [17,18] compared with zirconia (≈13 GPa) [19].
3. **Post-cutting procedure**: After each cycle, specimens were rinsed with distilled water, ultrasonically cleaned (SHB-1025; Sehansonic, Seoul, Republic of Korea) to remove debris, air-dried for 30 s, and re-weighed. Cutting efficiency was calculated as weight loss per unit time (mg/min).
4. **Sequential cutting**: Each diamond bur was used 10 consecutive cutting cycles on 10 separate specimens. Between cycles, lubricant (KaVo Quattrocare Plus; KaVo, Biberach, Germany) was applied for 1 s, and the handpiece was operated without load for 1 min to remove residual lubricant.
5. **Experimental repetition**: The full protocol was repeated with new burs (*n* = 10), producing 100 cutting measurements per specimen type and bur group.

#### 2.3.3. Evaluation Parameters

- Cutting efficiency: Weight difference before and after cutting (mg/min).
- Total cutting efficiency: Cumulative value from the 1st to 10th cycles, representing the overall cutting performance of each instrument.

### 2.4. Surface Characterization Methods

#### 2.4.1. Scanning Electron Microscopy (SEM)

A field-emission SEM (SUPRA 40 VP; Carl Zeiss, Oberkochen, Germany) was used to examine the morphology of diamond particles, metal-matrix bonding, and specimen surface characteristics.

- **Diamond bur analysis**: New (unused) and used (after 10 cycles) burs from both groups were observed at ×200 magnification. Qualitative observations included:
  ∘ Diamond particle size, shape, and morphology.
  ∘ Wear patterns and structural damage.
  ∘ Diamond particle dislodgement or attrition.
- **Cutting surface analysis**: Representative zirconia and enamel specimens were examined at ×100–×1000 magnification to assess:
  ∘ Microstructural features of the central cut area.
  ∘ Characteristics of the cut–uncut boundary.
  ∘ Presence of thermal damage or debris accumulation.

#### 2.4.2. Energy-Dispersive X-Ray Spectroscopy (EDS)

EDS analysis was performed to quantify compositional changes on bur surfaces before and after cutting. Measurements were taken at three regions (tip, middle, and base) for each bur. The analyzed elements were:

- Carbon (C): representing diamond particles.
- Nickel (Ni): representing the metal matrix.
- Oxygen (O): indicating possible oxidation or contamination.

New burs (*n* = 10 per group) were compared with those used after 10 cycles (*n* = 10 per group). The mean reduction rate of each element was calculated to estimate the degree of diamond particle loss or metal matrix degradation.

### 2.5. Statistical Analysis

All statistical analyses were performed using IBM SPSS Statistics v30.0 (IBM Corp., Armonk, NY, USA). Cutting efficiency values were expressed as mean ± standard deviation (mg/min). Comparisons between the natural diamond bur and synthetic diamond bur groups were made using the non-parametric Mann–Whitney U test for each cutting cycle. The significance level was set at α = 0.05.

The sample size (*n* = 10 burs per group, with each bur performing 10 cutting cycles on 10 separate specimens) was determined based on previous studies of diamond bur cutting efficiency [7,8,11,12], which demonstrated that this sample size provides adequate statistical power to detect clinically meaningful differences in cutting performance. Each bur group thus generated 100 cutting measurements (10 burs × 10 cycles), yielding sufficient data for robust statistical analysis. Preliminary power analysis indicated that *n* = 10 would provide >80% power to detect a 20% difference in cutting efficiency at α = 0.05.

## 3. Results

### 3.1. Cutting Efficiency Experiment

#### 3.1.1. Zirconia Block Cutting Efficiency

Cutting Efficiency by Number of Cuts

**Scheme 1:**
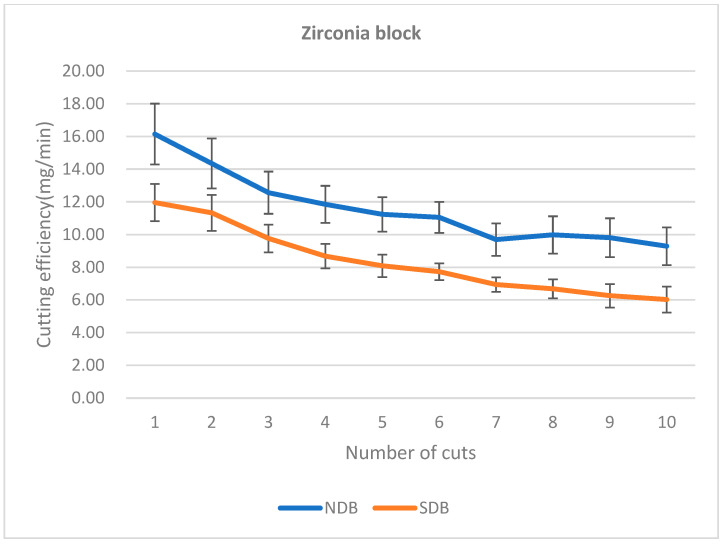
Mean and standard deviation of cutting efficiency according to number of cuts for zirconia blocks. NDB (blue), SDB (orange).

Natural diamond bur (NDB) showed statistically significantly higher cutting efficiency than synthetic diamond bur (SDB) at all cutting cycles (*p* < 0.05) (Table 1, Scheme 1). In the first cut, NDB showed a cutting efficiency of 16.15 ± 4.38 mg while SDB showed 11.96 ± 1.94 mg, which was a statistically significant difference (*p* = 0.011). In the second cut, NDB showed 14.35 ± 3.64 mg and SDB showed 11.33 ± 2.43 mg, also showing a significant difference (*p* = 0.035).

As cutting progressed, both groups showed a tendency for cutting efficiency to gradually decrease. In the middle cutting stages of the fifth and sixth cuts, NDB showed 11.24 ± 3.09 mg and 11.05 ± 2.91 mg, respectively, while SDB showed 8.09 ± 2.21 mg and 7.73 ± 1.68 mg, with significant differences continuing (*p* = 0.019, *p* = 0.005). This trend was maintained in the later cutting stages, with NDB recording 9.29 ± 4.01 mg and SDB recording 6.03 ± 2.47 mg in the tenth cut (*p* = 0.029).

Overall, NDB decreased by 42.5% in the tenth cut compared to the first cut, while SDB decreased by 49.6%, showing a greater reduction. This suggests that NDB maintains relatively stable cutting performance even with repeated use, indicating superior durability compared to SDB.

Total Cutting Efficiency

In terms of total cutting efficiency, which represents the total amount of material removed during 10 cuts, NDB showed 115.97 ± 2.22 mg, which was statistically significantly higher than SDB’s 83.46 ± 2.08 mg (*p* < 0.05). This means that NDB can remove approximately 38.9% more zirconia material under the same conditions.

Cutting Efficiency Reduction Rate

When the reduction rate of the tenth cutting efficiency was calculated based on the first cutting efficiency, NDB showed a reduction rate of 40.00 ± 13.66%, while SDB showed a reduction rate of 49.68 ± 18.97%. Although SDB showed a higher reduction rate, both groups showed large standard deviations, confirming considerable inter-individual variation. This suggests that SDB’s cutting performance tends to deteriorate more rapidly with repeated use.

#### 3.1.2. Natural Tooth Cutting Efficiency

**Scheme 2:**
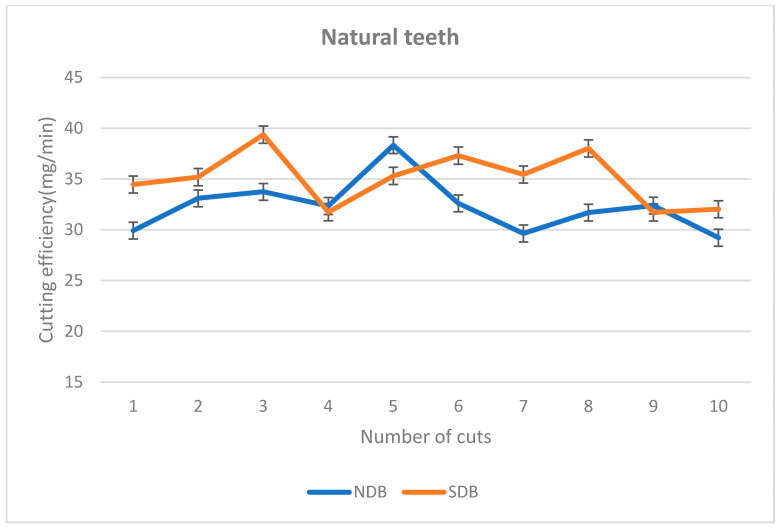
Mean and standard deviation of cutting efficiency according to number of cuts for natural teeth. NDB (blue), SDB (orange).

The mean ± standard deviation of cutting efficiency, total cutting efficiency, and cutting efficiency reduction rate according to the number of cuts for natural teeth are shown in Table 2, Scheme 2.

Cutting Efficiency by Number of Cuts

In natural tooth cutting, NDB and SDB showed no statistically significant differences at all cutting cycles (*p* > 0.05). In the first cut, NDB showed a cutting efficiency of 14.96 ± 5.48 mg and SDB showed 17.23 ± 5.92 mg, with no significant difference between the two groups (*p* = 0.529). In the second cut, they recorded 16.55 ± 5.91 mg and 17.59 ± 6.91 mg, respectively (*p* = 0.853).

As cutting progressed, no significant differences were observed between the two groups. In the fifth cut, NDB showed the highest value at 19.16 ± 8.73 mg, but there was no statistical difference from SDB’s 17.65 ± 6.88 mg (*p* = 0.853). In the later cutting stages of the eighth and ninth cuts, NDB showed 15.85 ± 5.02 mg and 16.19 ± 4.82 mg, while SDB showed 19.01 ± 7.64 mg and 15.86 ± 5.41 mg (*p* = 0.353, *p* = 0.912), and in the tenth cut, they recorded 14.61 ± 3.29 mg and 16.01 ± 5.82 mg, respectively (*p* = 0.579).

Notably, neither group showed a consistent decreasing trend even as the number of cuts increased, and cutting efficiency showed a fluctuating pattern by cycle. This is thought to be due to the relatively low hardness of natural tooth enamel and its non-uniform structural characteristics affecting cutting efficiency.

Total Cutting Efficiency

During 10 natural tooth cuts, NDB’s total cutting efficiency was 161.48 ± 5.27 mg and SDB’s was 175.27 ± 6.02 mg. Although SDB showed approximately 8.5% higher values, this was not a statistically significant difference (*p* > 0.05). This suggests that there is no difference in overall cutting amount between the two bur types in natural tooth cutting.

Cutting Efficiency Reduction Rate

The cutting efficiency reduction rate of the tenth cut compared to the first cut was 2.34% for NDB and 7.08% for SDB. These are significantly lower values compared to the reduction rates in zirconia block cutting (NDB 40.00%, SDB 49.68%).

These results clearly show that natural tooth cutting causes much less bur wear due to repeated use compared to zirconia block cutting. Both NDB and SDB maintained most of their initial cutting efficiency even after 10 repeated uses in natural tooth cutting.

### 3.2. Surface Characterization

#### 3.2.1. Surface Characteristics of Diamond Burs

The changes in elemental composition of diamond burs before and after cutting, as analyzed by EDS, are presented in Table 3.

Carbon Content Changes

The carbon content of NDB decreased from 65.676 ± 2.045 wt% (new) to 63.988 ± 7.504 wt% (used), showing a reduction rate of 2.494 ± 11.844%. In contrast, SDB exhibited a decrease from 77.845 ± 2.688 wt% (new) to 67.845 ± 8.662 wt% (used), demonstrating a reduction rate of 13.461 ± 9.607%. Although the initial carbon content of SDB was approximately 18.5% higher than that of NDB, the difference between the two groups narrowed to approximately 6.0% after use.

Nickel Content Changes

The nickel content of NDB showed a slight decrease from 32.463 ± 2.040 wt% to 31.58 ± 6.673 wt%; however, the negative reduction rate (−150.359 ± 313.956%) and large standard deviation indicate that nickel content increased in some measurement locations. SDB showed a distinct increase from 18.816 ± 2.956 wt% to 25.028 ± 9.076 wt%, corresponding to a negative reduction rate of −141.335 ± 344.843%.

Oxygen Content Changes

Oxygen content increased in both groups after use. NDB showed an increase from 1.860 ± 0.440 wt% to 4.432 ± 5.047 wt%, while SDB exhibited an increase from 3.339 ± 0.680 wt% to 7.477 ± 10.687 wt%.

Standard Deviation Changes

The standard deviations of the measured values increased after use. For SDB, the standard deviation of carbon content increased from 2.688 to 8.662, nickel content from 2.956 to 9.076, and oxygen content from 0.680 to 10.687. NDB also showed increased standard deviations after use, although to a lesser extent than SDB.

#### 3.2.2. Cutting Surface Characteristics

The microstructure of specimen surfaces after cutting was analyzed by FE-SEM.

**Figure 2:**
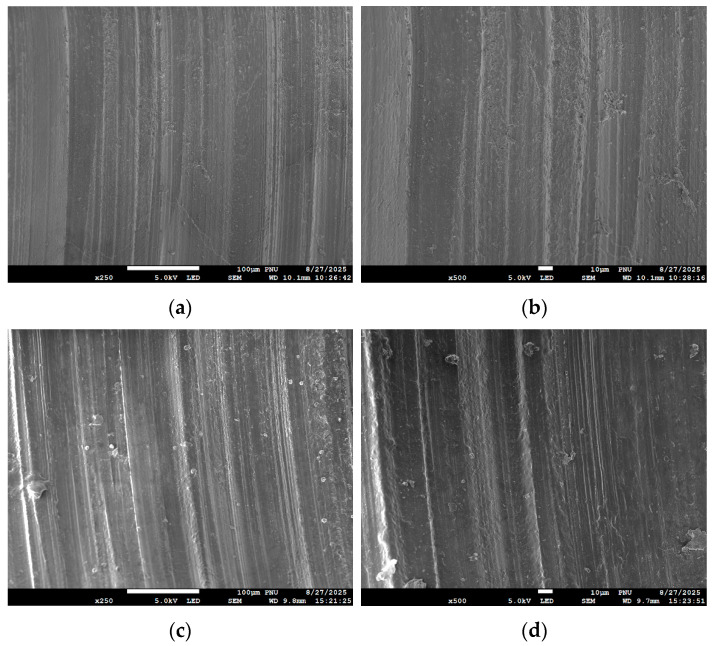
FE-SEM images of natural tooth specimen surfaces. (**a**,**b**) Center of natural tooth cutting surface cut with NDB (original magnification ×250, 500). (**c**,**d**) Center of natural tooth cutting surface cut with SDB. NDB cutting surfaces showed smaller scratch grooves and smoother surfaces compared to SDB cutting surfaces.

Natural tooth cutting surface: Natural tooth specimens were cut for 30 s. In the control group, the natural enamel prism structure without cutting was observed. The SDB group showed distinct scratch grooves and rough surfaces, while the NDB group showed smaller scratch grooves and smoother surfaces compared to the SDB group (Figure 2).

**Figure 3:**
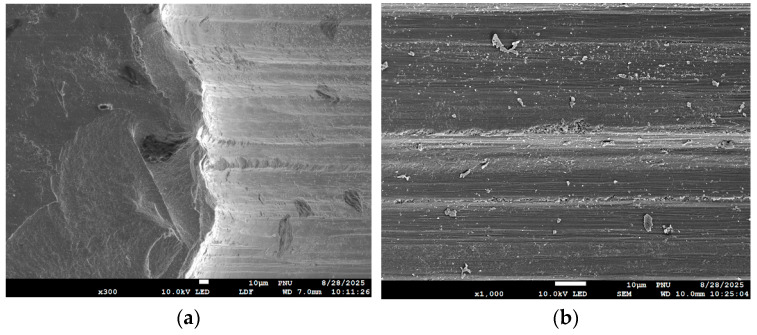

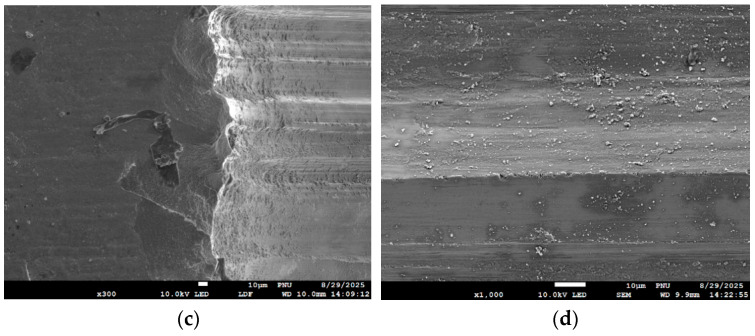
FE-SEM images of zirconia specimen surfaces. (**a**) Boundary of zirconia cutting surface cut with NDB (original magnification ×300). (**b**) Center of zirconia cutting surface cut with NDB (original magnification ×1000). (**c**) Boundary of zirconia cutting surface cut with SDB (original magnification ×300). (**d**) Center of zirconia cutting surface cut with SDB (original magnification ×1000). NDB cutting surfaces show similar surface morphology to SDB cutting surfaces.

Zirconia block cutting surface: Zirconia specimens were cut for 1 min. Similar cutting patterns were observed in both NDB and SDB groups, with scratch grooves from mechanical cutting appearing in both groups, and no distinct differences were seen in surface morphology (Figure 3).

## 4. Discussion

In this study, natural diamond burs exhibited significantly higher cutting efficiency than synthetic diamond burs across all zirconia cutting cycles (*p* < 0.05), with a total efficiency approximately 38.9% greater. This difference appears to originate from the intrinsic physical and chemical properties of the diamond particles and their bonding characteristics. Natural diamonds possess a uniform crystal structure and minimal impurities due to their natural formation under extreme conditions over long periods [13]. Given zirconia’s high Vickers hardness (≈13 GPa) [19], substantial mechanical stress was exerted on the cutting surface, and thus, the superior crystal integrity and bonding strength of natural diamond particles likely contributed to their enhanced performance [14]. While high-quality CVD synthetic diamonds can exhibit purity and uniformity superior to many natural diamonds, the synthetic burs evaluated here may have been affected by incomplete sintering, graphitization, or metallic impurities from the HPHT process. This emphasizes the importance of rigorous quality control in synthetic diamond manufacturing for dental applications. The superior performance of natural diamond burs in this study should be interpreted in the context of the specific commercial products tested, rather than as a general superiority of all natural diamonds over all synthetic diamonds. From a methodological standpoint, the same handpiece was used throughout the experiment to maintain consistency; any potential performance changes due to cumulative use would have affected all groups equally, thereby ensuring the validity of comparative results.

The plating technique used for the burs may have contributed to performance differences. Natural diamond burs were manufactured with nickel sulfamate electroless plating, which is known to yield a uniform, low-stress coating that promotes strong retention of diamond particles. In contrast, the synthetic diamond burs evaluated in this study employed conventional nickel electroplating, which may exhibit greater residual stress and weaker adhesion. Correspondingly, natural diamond burs showed smaller efficiency reduction with repeated use (40.00%) compared to synthetic diamond burs (49.68%), which may indicate better particle retention. These findings differ somewhat from earlier reports that found no improvement with zirconia-specific burs [9], possibly due to differences in study design, diamond quality, and plating techniques evaluated.

In natural tooth cutting, no statistically significant difference was observed between the two bur types (*p* > 0.05). Because the hardness of enamel and dentin (3–4 GPa) [17,18] is far lower than that of zirconia, both burs were able to cut effectively, with minimal reduction in efficiency (2.34–7.08%). The total cutting efficiency for natural teeth was higher than that for zirconia, indicating that substrate hardness has a major influence on cutting performance even after accounting for different cutting durations. Interestingly, synthetic diamond burs performed 110% more efficiently on natural teeth than on zirconia, confirming their adequate suitability for low-hardness materials.

**Figure 4:**
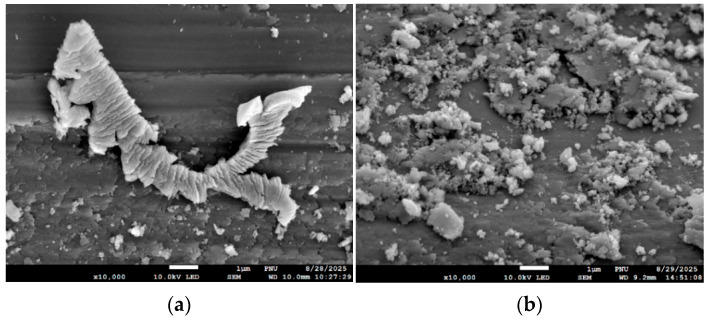
FE-SEM images of zirconia specimen surfaces. (**a**) Plate-like zirconia particles from NDB (original magnification ×10,000). (**b**) Particulate and curled plate-like zirconia particles from SDB (original magnification ×10,000).

Surface analysis results supported these findings. SEM observations revealed that natural diamond burs produced finer scratches and smoother surfaces than synthetic diamond burs on enamel, likely due to uniform particle morphology and stronger matrix bonding In zirconia specimens, debris morphology showed predominantly plate-like particles from NDB cutting, while SDB produced a mixture of particulate and curled plate-like particles (Figure 4), suggesting differences in cutting mechanisms (ductile versus brittle cutting modes). Previous literature has reported that ductile cutting can generate compressive stress layers that may resist low-temperature degradation (LTD), whereas brittle cutting may promote microcracks that can accelerate moisture penetration and structural weakening [20,21,22,23,24]. Thus, natural diamond burs may enhance not only cutting efficiency but also the long-term surface stability of zirconia restorations when performing occlusal adjustments, margin corrections, or endodontic access through existing prostheses [25,26,27]. However, confirmation of these effects in our specimens would require additional mechanical testing or aging experiments beyond the scope of this study.

Both bur types showed progressive declines in efficiency with repeated zirconia cutting [8] though the reduction was greater for synthetic burs (49.68%) than for natural burs (40.00%). This decrease is attributed to wear, particle loss, and deformation of the metal matrix.

The EDS analysis provided insights into surface compositional changes, though it is important to note that these measurements reflect surface signal variation and heterogeneity rather than bulk chemical composition. SDB exhibited approximately 5.4 times greater carbon reduction compared to NDB, directly correlating with the observed differences in cutting efficiency reduction rates. This suggests that loss of diamond particles is the primary mechanism underlying cutting performance degradation. Interestingly, despite SDB’s initially higher carbon content, its total cutting efficiency remained substantially lower, suggesting that particle bonding strength and interfacial characteristics are more critical determinants than simply the quantity of diamond particles present.

The observed increase in nickel content, particularly in SDB, may suggest progressive exposure of the nickel matrix following diamond particle dislodgement. However, given the high variability and the absence of independent confirmation through other analytical methods, this interpretation should be considered tentative. Since the nickel matrix possesses significantly lower hardness compared to diamond particles, exposure of the metal matrix cannot effectively cut zirconia, contributing to SDB’s accelerated efficiency loss.

The increase in oxygen content observed in both groups may reflect surface phenomena during zirconia cutting, potentially including oxidation of exposed metal surfaces, mechanical embedding of zirconia debris, or measurement variation from heterogeneous wear patterns. The higher variability in SDB suggests more active surface changes and heterogeneous debris accumulation. Further investigation with complementary analytical techniques would be needed to confirm the specific mechanisms.

The substantial increase in measurement standard deviations after use reflects heterogeneous surface wear patterns. The greater variability observed in SDB indicates uneven diamond particle retention and irregular debris accumulation across the bur surface, which may translate to clinically unpredictable cutting performance. The relatively smaller variability in NDB suggests stronger and more uniform bonding, contributing to more consistent cutting performance even with repeated use.

These compositional findings also raise important questions about quality variability in commercial synthetic diamond burs. As discussed in the introduction, synthetic diamonds can exhibit degraded mechanical properties due to incomplete sintering, graphitization, or metallic impurities from the HPHT process [14]. SDB’s lower cutting efficiency despite initially higher carbon content suggests such quality inconsistencies may compromise performance predictability.

Based on these in vitro findings, natural diamond burs may offer advantages for zirconia adjustments in terms of cutting efficiency and wear resistance. However, clinical validation is recommended before definitive recommendations can be made, as in vivo conditions may differ significantly from the controlled laboratory environment employed in this study. From a practical standpoint, maintaining standardized handpiece conditions (200,000 rpm with adequate air pressure and continuous water cooling) is important when using diamond burs to ensure consistent performance.

This study has several limitations. First, as an in vitro experiment, it could not fully reproduce clinical conditions including operator pressure variation, bur angulation, and the presence of saliva and soft tissues. Second, only 10 cutting cycles were evaluated, only diamond burs from a single manufacturer were tested, and only enamel was examined for natural teeth, limiting generalizability. Third, substantial interindividual variation exists among natural teeth, and cutting resistance likely differed according to enamel thickness, curvature, and prism orientation; this variability may explain the larger standard deviation observed in the cutting efficiency of natural teeth compared with zirconia. Fourth, while continuous water cooling was employed, direct thermal monitoring and quantitative surface roughness analysis were not performed. Despite these limitations, this study provides valuable controlled data demonstrating significant performance differences between natural and synthetic diamond burs under standardized conditions.

## 5. Conclusions

In this in vitro study, natural diamond burs demonstrated significantly higher cutting efficiency than synthetic diamond burs on zirconia, while both types showed comparable performance on natural teeth. Surface characterization revealed that natural diamond burs produced smoother enamel surfaces. SEM observations also suggested more favorable surface characteristics on zirconia with reduced microcrack propagation. These results indicate that bur selection should be guided by substrate hardness: natural diamond burs are recommended for high-hardness materials such as zirconia, whereas either type may be used for natural tooth preparation. Further investigation incorporating multiple manufacturers and clinical validation is warranted.

## Figures and Tables

**Table 1:** Mean ± standard deviation of cutting efficiency, total cutting efficiency, and cutting efficiency reduction rate according to number of cuts for zirconia blocks.

Number of Cuts	NDB (mg)	SDB (mg)	*p*-Value
1st cycle	16.15 ±4.38 ^A^	11.96 ± 1.94 ^B^	0.011 *
2nd cycle	14.35 ± 3.64 ^A^	11.33 ± 2.43 ^B^	0.035 *
3rd cycle	12.56 ± 3.34 ^A^	9.76 ± 2.02 ^B^	0.043 *
4th cycle	11.85 ± 3.08 ^A^	8.68 ± 2.16 ^B^	0.019 *
5th cycle	11.24 ± 3.09 ^A^	8.09 ± 2.21 ^B^	0.019 *
6th cycle	11.05 ± 2.91 ^A^	7.73 ± 1.68 ^B^	0.005 **
7th cycle	9.69 ± 3.37 ^A^	6.94 ± 1.56 ^B^	0.015 *
8th cycle	9.98 ± 3.93 ^A^	6.68 ± 2.00 ^B^	0.019 *
9th cycle	9.81 ± 4.13 ^A^	6.26 ± 2.35 ^B^	0.023 *
10th cycle	9.29 ± 4.01 ^A^	6.03 ± 2.47 ^B^	0.029 *
Total cutting efficiency	115.97 ± 2.22 ^A^	83.46 ± 2.08 ^B^	<0.05
Reduction rate	40.00 ± 13.66%	49.68 ± 18.97%	-

NDB, Natural diamond bur; SDB, Synthetic diamond bur. Different uppercase superscripts in each row indicate statistically significant differences (* *p* < 0.05, ** *p* < 0.01, Mann–Whitney U test).

**Table 2:** Mean ± standard deviation of cutting efficiency, total cutting efficiency, and cutting efficiency reduction rate according to number of cuts for natural teeth.

Number of Cuts	NDB (mg)	SDB (mg)	*p*-Value
1st cycle	14.96 ± 5.48	17.23 ± 5.92	0.529
2nd cycle	16.55 ± 5.91	17.59 ± 6.91	0.853
3rd cycle	16.87 ± 3.67	19.68 ± 5.63	0.436
4th cycle	16.17 ± 6.47	15.87 ± 4.98	0.853
5th cycle	19.16 ± 8.73	17.65 ± 6.88	0.853
6th cycle	16.30 ± 3.92	18.65 ± 5.37	0.143
7th cycle	14.82 ± 2.45	17.72 ± 5.02	0.165
8th cycle	15.85 ± 5.02	19.01 ± 7.64	0.353
9th cycle	16.19 ± 4.82	15.86 ± 5.41	0.912
10th cycle	14.61 ± 3.29	16.01 ± 5.82	0.579
Total cutting efficiency	161.48 ± 5.27	175.27 ± 6.02	>0.05
Reduction rate	2.34%	7.08%	-

NDB, Natural diamond bur; SDB, Synthetic diamond bur. No statistically significant differences were observed at any cycle (*p* > 0.05, Mann–Whitney U test).

**Table 3:** Changes in elemental composition of diamond burs before and after cutting (wt%). NDB, Natural diamond bur; SDB, Synthetic diamond bur. Negative values in reduction (%) indicate an increase. Red, green, and blue spheres represent carbon, nickel, and oxygen atoms, respectively.

	SEM	EDS Layered Image	Map Sum Spectrum	
NDB new	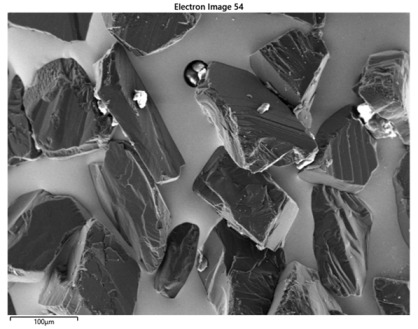	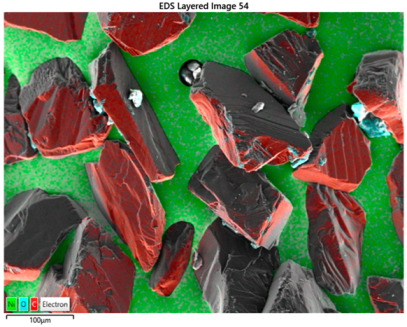	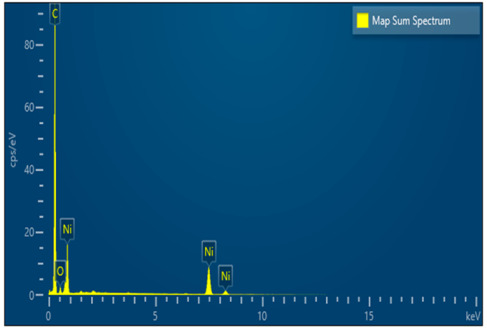	
NDB used	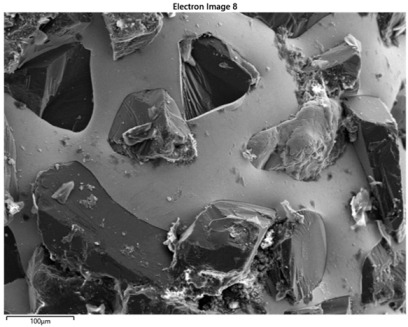	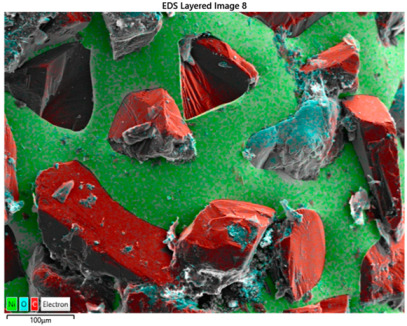	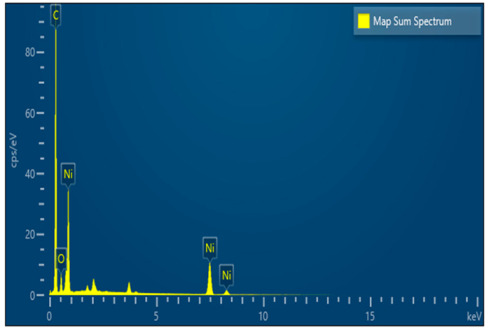	
SDB new	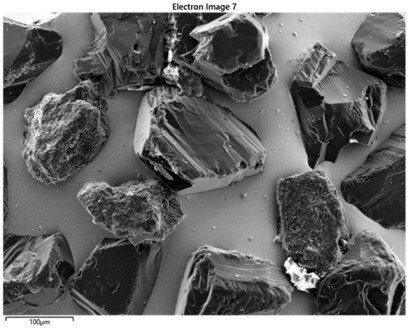	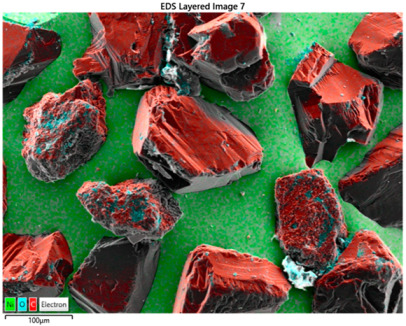	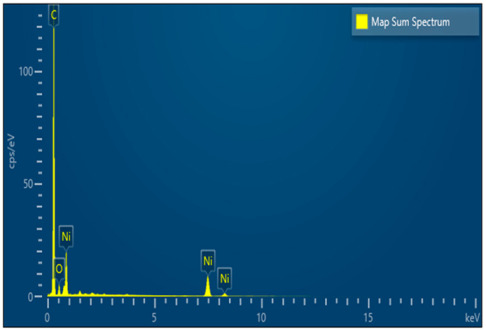	
SDB used	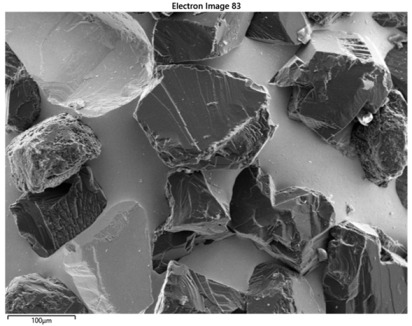	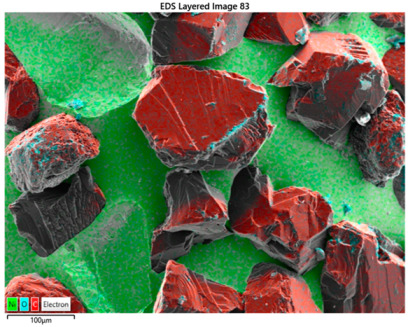	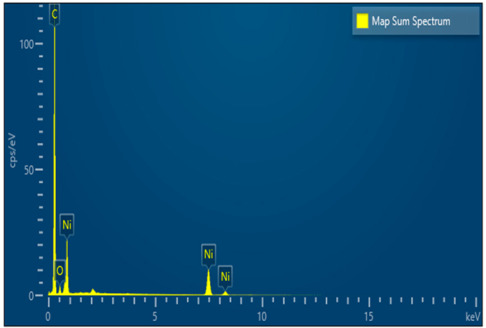	
Group	Condition	Carbon (wt%, mean ± SD)	Nickel (wt%, mean ± SD)	Oxygen (wt%, mean ± SD)
NDB	New	65.676 ± 2.045	32.463 ± 2.040	1.860 ± 0.440
NDB	Used	63.988 ± 7.504	31.58 ± 6.673	4.432 ± 5.047
NDB	Reduction (%)	2.494 ± 11.844	−150.359 ± 313.956	2.511 ± 19.978
SDB	New	77.845 ± 2.688	18.816 ± 2.956	3.339 ± 0.680
SDB	Used	67.845 ± 8.662	25.028 ± 9.076	7.477 ± 10.687
SDB	Reduction (%)	13.461 ± 9.607	−141.335 ± 344.843	−33.3195 ± 40.643

## Data Availability

The original contributions presented in this study are included in the article. Further inquiries can be directed to the corresponding authors.

## Abbreviations

The following abbreviations are used in this manuscript:

NDB	Natural diamond burs
SDB	Synthetic diamond burs
EDS	Energy-Dispersive X-ray Spectroscopy
FE-SEM	Field-Emission Scanning Electron Microscopy
HPHT	High-Pressure High-Temperature
CVD	Chemical Vapor Deposition
LTD	Low-Temperature Degradation
